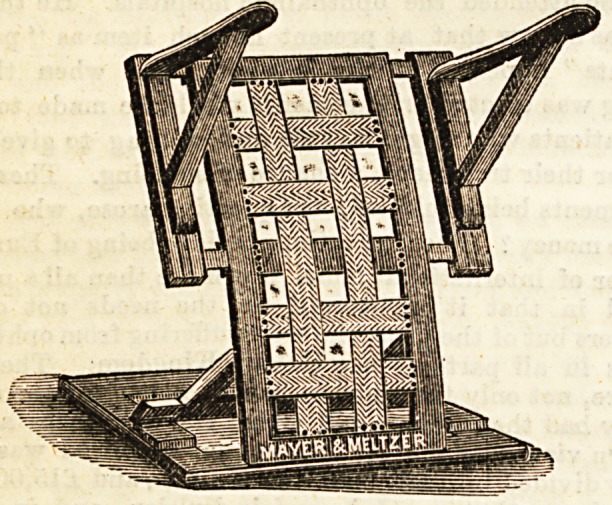# Bed Rests

**Published:** 1892-05-28

**Authors:** 


### FURNITURE AND FITTINGS.

### BED RESTS.

No. 1.—Bed Rest with Foot Support.

In the bed rest as shown in our illustration we have an article that all engaged in the nursing of the sick must have felt to be a great want. The ordinary bed rests prove one of the least restful of all contrivances from the nurse's point of view. Constant adjustment is required, owing to the patient slipping out of position. The simple arrangement of a foot support which is easy of regulation supplies all that is required. The rests have been awarded certificates of merit and honourable mention at the International Medical and Sanitary Congress and the National Health Society's Exhibition. The rest was invented by Mr. Newton H. Nixon, and it has been for some time in use at University College Hospital. It can be procured from the manufacturers, Messrs. Mayer and Metzler, London.

No. 2.—Bed Rest with Sliding Arms.

Another improvement on the old bed rests is the bed rest with sliding arms. It also has the advantage of assisting the patient to retain a desired position, and admits of a variation without undesirable shifting, as the arms slide vertically and horizontally, and are on hinges fitted to a rack, so that the angle may be altered to suit the position of the patient.

This rest forms perhaps a greater support than any to the invalid when in a sitting posture. It is made by the same firm as mentioned above, and was contrived also by Mr. Newton H. Nixon.

## Figures and Tables

**Figure f1:**
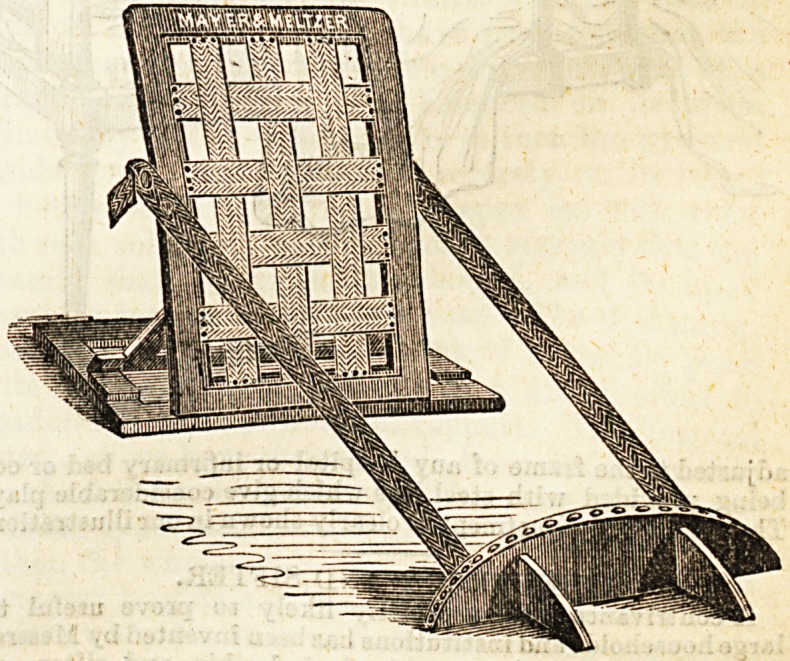


**Figure f2:**